# M_4_X_3_ MXenes: Application in Energy Storage Devices

**DOI:** 10.1007/s40820-024-01418-0

**Published:** 2024-06-14

**Authors:** Iftikhar Hussain, Waqas Ul Arifeen, Shahid Ali Khan, Sikandar Aftab, Muhammad Sufyan Javed, Sajjad Hussain, Muhammad Ahmad, Xi Chen, Jiyun Zhao, P. Rosaiah, Khaled Fahmi Fawy, Adnan Younis, Sumanta Sahoo, Kaili Zhang

**Affiliations:** 1 Department of Mechanical Engineering, City University of Hong Kong, 83 Tat Chee Avenue, Kowloon Tong, Hong Kong People’s Republic of China; 2 School of Mechanical Engineering, Yeungnam University, Daehak-ro, Gyeongsan-si, Gyeongbuk-do 38541 South Korea; 3 Department of Semiconductor Systems Engineering and Clean Energy, Sejong University, Seoul, 05006 Republic of Korea; 4 School of Physical Science and Technology, Lanzhou University, Lanzhou, 730000 People’s Republic of China; 5 Department of Nanotechnology and Advanced Materials Engineering, Sejong University, Seoul, 05006 Republic of Korea; 6 Department of Physics, Saveetha School of Engineering, Saveetha Institute of Medical and Technical Sciences (SIMATS), Thandalam, Chennai, 602 105 India; 7 Department of Chemistry, Faculty of Science, King Khalid University, P.O. Box 9004, 61413 Abha, Saudi Arabia; 8 Department of Physics, College of Science, United Arab Emirates University, P.O. Box 15551, Al-Ain, United Arab Emirates; 9 School of Chemical Engineering, Yeungnam University, Gyeongsan, Gyeongbuk 38541 South Korea

**Keywords:** MXene, M_4_X_3_ MXenes, 2D materials, Energy storage, Properties

## Abstract

MXene has garnered widespread recognition in the scientific community due to its remarkable properties, including excellent thermal stability, high conductivity, good hydrophilicity and dispersibility, easy processability, tunable surface properties, and admirable flexibility. MXenes have been categorized into different families based on the number of M and X layers in M_n+1_X_n_, such as M_2_X, M_3_X_2_, M_4_X_3_, and, recently, M_5_X_4_. Among these families, M_2_X and M_3_X_2_, particularly Ti_3_C_2_, have been greatly explored while limited studies have been given to M_5_X_4_ MXene synthesis. Meanwhile, studies on the M_4_X_3_ MXene family have developed recently, hence, demanding a compilation of evaluated studies. Herein, this review provides a systematic overview of the latest advancements in M_4_X_3_ MXenes, focusing on their properties and applications in energy storage devices. The objective of this review is to provide guidance to researchers on fostering M_4_X_3_ MXene-based nanomaterials, not only for energy storage devices but also for broader applications.

## Highlights

- A systematic overview of the latest advancements in M_4_X_3_ MXenes is dicussed.
- The detailed properties of MXene are summarized.
- M_4_X_3_ MXenes are explored as an electrode material.

## Graphical abstract

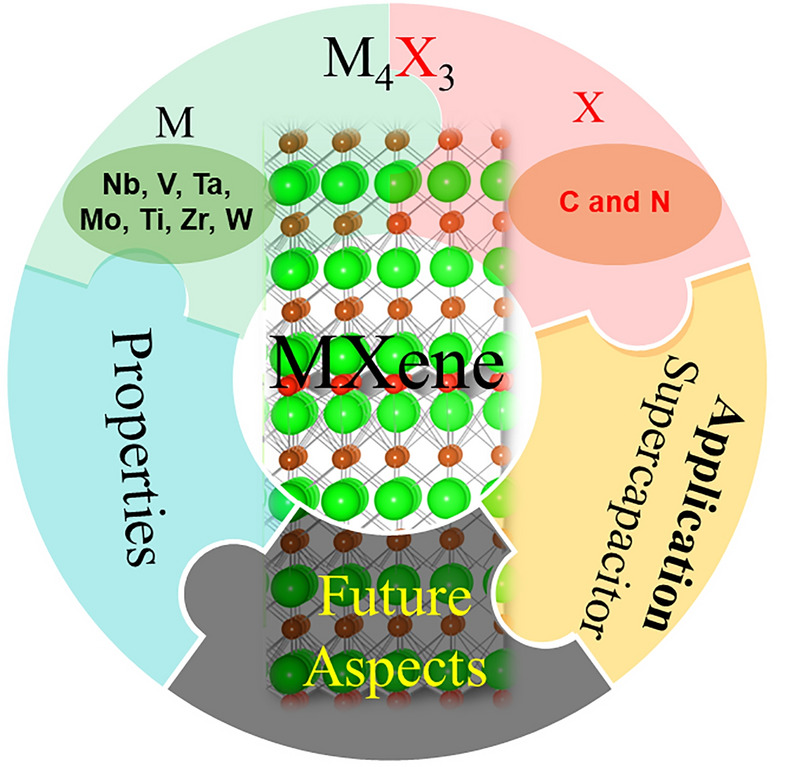

## Introduction

Two-dimensional (2D) materials offer superior electronic structures, high specific surface areas, and other properties compared to their bulk counterparts [1, 2]. Graphene has been extensively studied as a prominent 2D material. There is a growing interest in exploring other 2D materials such as metal oxides and hydroxides, metal dichalcogenides, and hexagonal boron nitride [3–7]. Each 2D materials have been studied in various applications due to their unique properties [4, 8]. These 2D material properties make them suitable for a variety of applications, including; solar cells and touch screens [9], transistors [10], lasers [11], and biosensors [12] compared to the bulk counterparts of these 2D materials. By increasing complexity and diversity, it becomes possible to achieve exceptional combinations of properties. Further, 2D materials present convenient nanoscale building blocks for assembling architectures. In 2011, a significant addition to the family of 2D materials was made with the discovery of MXenes [13]. MXenes encompass an extensive array of two-dimensional (2D) materials, consisting of transition-metal carbides, nitrides, and carbonitrides [14–17].

Among various 2D materials, MXenes represent a diverse family of 2D materials encompassing an infinite number of solid-solution MXenes, numerous compositions predicted through computational methods, and over 50 stoichiometric MXenes that have been successfully synthesized [18]. MXenes exhibit a unique combination of optical, electronic, mechanical, and colloidal properties, making them highly versatile materials [19–22]. To be specific, this class of materials display superior conductivity, enhanced surface area, improved electrochemical performance, improved hydrophilicity, and better chemical stability than majority of its conventional 2D counterparts [23–25]. By etching the A element from MAX (M is an early transition metal; A is an element from group 13 or 14 of the periodic table, commonly Al or Si precursor phase, 2D layers are produced called MXene [26–29]. The general formula for the MXene is M_*n*+*1*_X_*n*_T_*x*_, where M is a transition metal, X is C or N, n = 1–4, and T_*x*_ is the surface termination. The typical solution-processed synthesis techniques result in T_*x*_ being a non-uniform mixture of –OH, –F, and =O [30]. MXenes have attracted an ever-increasing attention from different research communities, including electrochemistry [31], electromagnetic wave absorption/shielding [32], catalysis [3], sensing [33], biomedicine [34], energy harvesting [35], and so on. This is because of their diverse and adaptable chemical, optical, electrical, mechanical, and optical properties [36]. It has also been studied that the structure and composition of MXene are crucial in determining these properties [37].

The MXene family is capable of producing materials with 2, 3, 4, or 5 atomic layers of transition metal. The final MXene structure is dependent on its MAX phase precursor(s) [6]. MXene compositions such as V_2_CT_*x*_ [38]_*,*_ Nb_2_C [39], Ti_3_C_2_T_*x*_ [40], V_4_C_3_T_*x*_ [38], and Mo_4_VC_4_T_*x*_ [41] have been reported. Among all, Ti_3_C_2_T_*x*_ has been widely explored and studied for different applications [6]. The type of MAX phase, synthesis conditions (temperature, concentration, and etching time), and choice of etching solution all contribute to the exfoliation degree and subsequent characteristics of MXenes [42]. Careful control and optimization of these factors are essential for tailoring the properties of MXenes for specific applications [43]. Various synthesis methods such as HF etching [30, 44], acid/fluoride salt or hydro-fluoride etching for in-situ production of HF [45–48], electrochemical etching [49], alkali etching [50], molten salt etching [51], chemical vapor deposition, and atomic layer deposition [52] have been explored. These approaches will result in different MXene structures and surface chemical states, which will have an impact on the overall behavior and performance of MXenes, as discussed in detail previously [53]. Thus, by utilizing the appropriate synthesis process, researchers can maximize the potential of MXenes and tailor their properties to suit a wide range of application [54, 55].

MXenes can be classified into different families based on the number of atomic layers [18]. Each family of MXenes is particularly noteworthy depends on the applications and their properties. MXenes can be further categorized into two distinct types based on their transition-metal composition [56]. The first category is known as mono-transition-metal (Mono-M) MXenes, in which the M layers consist of a single type of transition metal. Examples of Mono-M MXenes include Ti_2_CT_*x*_, V_2_CT_*x*_, Ti_3_C_2_T_x_, Nb_4_C_3_T_x_, and others. The second category is referred to as double transition-metal (DTM) MXenes. DTM MXenes are composed of two different transition metals, such as Mo_2_Ti_2_C_3_T_*x*_, Mo_2_TiC_2_T_*x*_, and so on. DTM MXenes can also be classified based on their structure into two categories: ordered MXenes and solid-solution MXenes. Within the ordered MXenes, there are further classified into in-plane order and out-of-plane order [56].

**Fig. 1:**
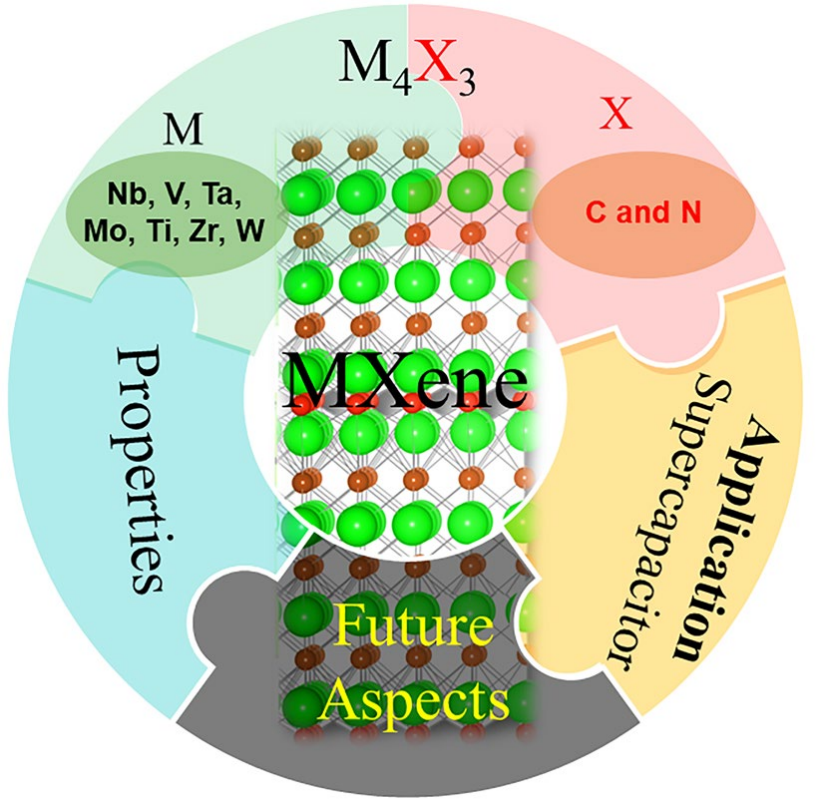
Overview schematic of the review focus areas

Number of reviews on MXenes, MXenes composites, DTM MXenes, etc. have already been published [5, 56–62] for various applications. It is highly desirable to describe this feature to better comprehend the development potential of MXene family based on the number of atomic layers they possess in the field of energy storage [63–66], specifically supercapacitors (SCs) [67–70]. M_3_X_2_ particularly Ti_3_C_2_ and M_2_X and have been greatly explored among different MXenes based on the number of atomic layers [71]. In comparison, the M_5_X_4_ and M_4_X_3_ MXenes have received relatively less attention and are more challenging to synthesize. As of now, only a limited number of M_5_X_4_ MXenes have been synthesized, which means that comprehensive compilation and understanding of these materials as a review will require more time and research efforts [18]. Herein, we focused a review on M_4_X_3_ MXenes. M_4_X_3_ MXenes display exceptional electronic, magnetic, electrochemical, optical, and mechanical properties, making them stand out in the realm of 2D materials [71]. Benefited by its structural features, such MXene displayed enhanced oxidation resistance and improved electrochemical activity than its other MXene counterparts. For example, a previous report already demonstrated higher capacitance for Ta_4_C_3_T_*x*_ MXene than Ti_3_C_2_T_*x*_ and Ti_2_CT_*x*_ MXenes [72]. Furthermore, V_4_C_3_T_*x*_ MXene also exhibited higher specific capacitance than several Ti_3_C_2_T_*x*_ MXene and related composites [68]. To date, there is currently no review specifically focused on M_4_X_3_ MXenes. This highlights the need for a comprehensive examination and analysis of the properties, applications, and potential future directions of M_4_X_3_ MXenes as shown in Fig. 1. Such a review would contribute significantly to the understanding and advancement of this unique class of 2D materials.

## Overview of Supercapacitors

Energy storage devices are the pioneer of modern electronics world. Among, SCs have been widely studied because of their improved electrical performance including fast charge/discharge ability, enhanced power density, and long cycle life [73–75]. Based on the energy storage mechanism, supercapacitors classified principally into three main classes: Electric double layer capacitors (EDLCs), pseudocapacitors or redox capacitors (PCs), and hybrid capacitors (HCs) [76–80]. EDLCs can store charges by non-faradaic electrochemical process i.e. electrostatically by forming electric double layer at electrode and electrolyte interface [81, 82]. An electrostatic charge storage is simple and fast as it stores charges on electrode surface without any charge transfer between the electrode and electrolyte ions. However, PCs store charges through Faradaic electrochemical process, wherein charge transfer occurs between the electrode and electrolyte. On the other hand, HCs following both the charge storage mechanism. While the carbon-based materials display the EDLC-type charge storage mechanism, the metal oxides generally follow the PC-type charge storage route [83, 84]. For achieving prominent electrochemical performance, any SC electrodes should have good conductivity, large surface area, and high porosity. MXene materials usually display PC-type charge storage mechanism [85–88]. For the common Ti_3_C_2_T_x_ MXene, the presence of water molecules between the layers plays significant role on activating the redox reactions of the Ti atoms [89]. To improving the capacitive performance, MXenes have been systematically integrated with other carbon materials [90–92], metal oxides [93, 94], metal sulfides, hydroxides etc.

## Expansion of the M_4_X_3_ MXene

**Fig. 2:**
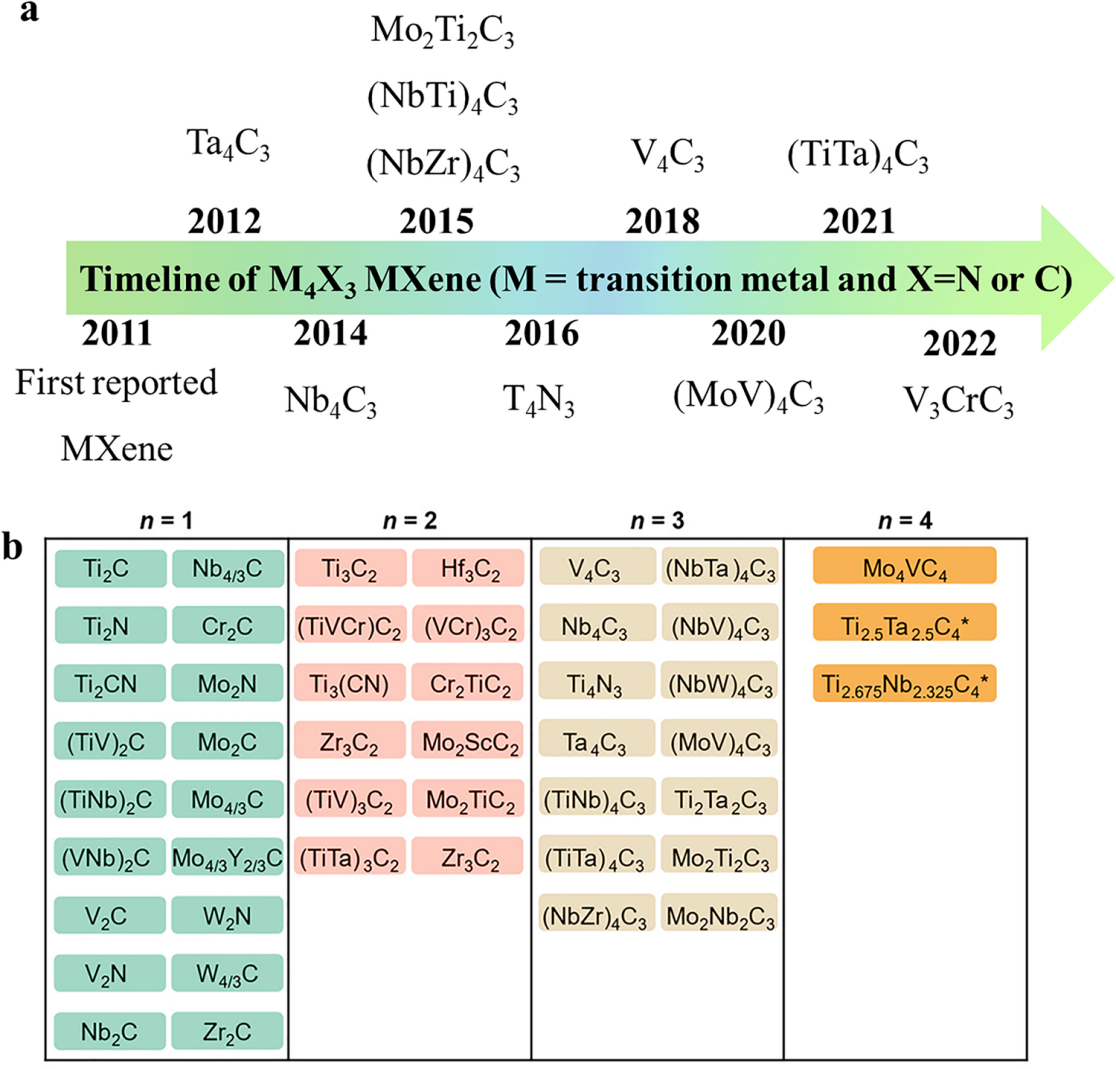
**a** A brief timeline of the progress in M_4_X_3_ MXenes. **b** Synthesized MXenes reported to date, including 18 M_2_X, 12 M_3_X_2_, 14 M_4_X_3_, and three M_5_X_4_. Reproduced with permission from Ref. [18]. Copyright 2023**,** American Chemical Society

Figure 2a illustrates the timeline of M_4_X_3_ MXenes, highlighting the substantial expansion of this MXene family since their discovery. New members have been added almost every year, showcasing the continuous growth of MXenes. However, when compared to M_2_X the number of publications on M_4_X_3_ MXenes low due to the complex synthesis approach. Nonetheless, there has been a recent increase in research interest towards M_4_X_3_ MXenes due to their fascinating properties. Ta_4_C_3_, Nb_4_C_3_, Mo_2_Ti_2_C_3_ (NbTi)_4_C_3_, (NbZr)_4_C_3_, T_4_N_3_, V_4_C_3_, (MoV)_4_C_3_, (TiTa)_4_C_3_, and V_3_CrC_3_ have been successfully synthesized and investigated for various applications [18]. These materials have exhibited intriguing physicochemical properties in various studies. Although M_4_X_3_ MXenes have received less attention from researchers compared to M_2_X and M_3_X_2_, Recent studies have revealed that M_4_X_3_ MXenes possess unique and compelling properties [68, 69, 95, 96]. This has sparked growing interest in exploring their potential applications and expanding our understanding of these materials. To date, over 50 stoichiometric MXenes have been experimentally synthesized in the lab [18]. These include various combinations of transition metals (M) and carbon/nitrogen (X) elements. Each stoichiometric MXene possesses distinct characteristics and potential applications. In addition to the experimentally synthesized MXenes, there are hundreds of MXene compositions that have been computationally predicted. These computationally predicted MXenes expand the possibilities for exploring novel materials with tailored properties. Downes et al. [18] summarized almost all the M_2_X, M_3_X_2_, M_4_X_3,_ and even the newly reported M_5_X_4_ MXenes as show in Fig. 2b. M_4_X_3_ MXene stands out from other MXenes due to its unique set of advantages, making it an attractive material for electrochemical energy storage devices. The Nb_4_C_3_T_*x*_ MXene demonstrated impressive performance for Li-ion batteries due to their excellent electronic conductivity and large interlayer spacing which accommodated more lithium ions [97]. Further, it has garnered increasing attention due to its exceptional electrical and mechanical properties [98]. These combined factors motivated researcher to study Nb_4_C_3_T_*x*_ for energy storage applications. For instance, V_4_C_3_ MXene possess greater interlayer spacing which facilitates the insertion and diffusion of ions during charging and discharging processes, leading to improved electrochemical performance. Furthermore, it also exhibits excellent structural durability, making it highly resistant to mechanical stress and deformation during battery operation. In addition to its mechanical properties and thermal stability, V_4_C_3_ MXene possesses tremendous metallic properties due to a narrow band gap at the Fermi level, making it highly conductive [99].

## Properties and Advantages of MXene Appropriate for SCs

**Fig. 3:**
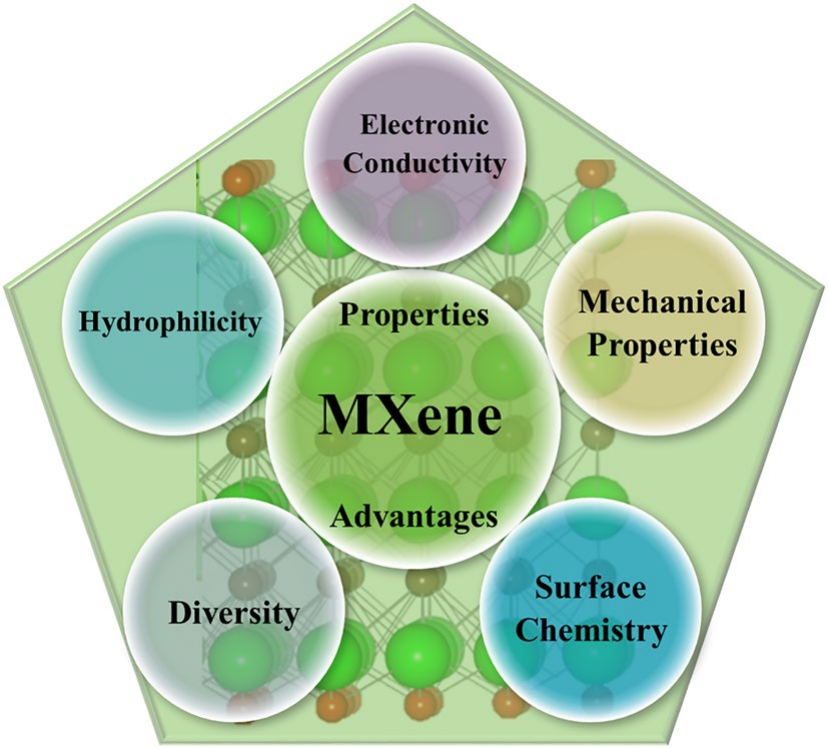
Unique properties of MXene materials

MXenes offer numerous excellent properties and advantages that make them highly attractive for various applications. Some key properties and advantages of MXenes are shown in Fig. 3**.**

### Electronic Conductivity

The electronic conductivity of MXenes is influenced by various factors, including their composition, structure, surface groups, preparation conditions, and post-etching treatments [4, 57]. Increasing the surface area of MXenes and reducing defects are effective approaches to improving their conductivity [26, 100, 101]. The excellent electrical conductivity enables MXenes to be utilized in a wide range of applications, including sensors, thermal heaters, electromagnetic interference shielding, and energy storage [5, 59]. In the context of energy storage, MXenes can also serve as current collectors, establishing a conductive framework that enhances the electronic conductivity of electrode materials. Furthermore, when building electrodes or current collectors, this high conductivity might eliminate the need for extra conductive materials and binders, simplifying the manufacturing process and reducing costs. This conductivity enhancement facilitates efficient charge transport within the electrode, leading to improved performance in energy storage devices [102, 103]. The high surface area of MXene allows for a higher quantity of active sites available for charging/discharging, resulting in enhanced energy storage capacity.

### Hydrophilicity

Hydrophilic surfaces have a strong affinity for water are preferred in the dispersion of water. The MXene ink need to be dispersed in different solvents for the fabrication of electrodes. The hydrophilicity of MXenes enable its compatibility with aqueous electrolytes. The stable hydrophilicity of MXene in aqueous solutions relies on the presence O and OH groups. The hydrophilic properties of MXene can be controlled by adjusting the surface terminations (OH, F, Cl, O) through specific synthesis techniques. Furthermore, the surface terminal groups of MXene play a significant role in determining the electrochemical performance of MXene-based electrodes for supercapacitors [104]. MXene can also be utilized as a current collector in other energy storage devices such as aqueous Zn-batteries. It has the possibility to achieve a uniform flux of Zn ions. This promotes homogenized Zn deposition, leading to improved battery performance [7]. Moreover, the hydrophilic properties of MXenes also make it interact favorably with polymer matrices. This beneficial interaction is particularly advantageous when MXenes are used in composite materials. Thus, the hydrophilic nature of MXenes contributes to their successful integration and utilization in composite materials [105].

### Mechanical Properties

MXenes are highly promising materials for various devices due to their flexible nature and unique 2D-lamellar structure. MXene possesses outstanding mechanical properties attributed to the high binding strength of M–C/M–N bonds. MXenes have high tensile strength and elastic modulus. Even when MXene films are folded or bent into different shapes, they demonstrate outstanding mechanical flexibility without any noticeable damage [70, 106]. The strength of M–X bonds in MXenes can be assessed by examining their bond stiffness, which is determined by the bond energy and the length of the M–X bond. The bond stiffness provides insights into the theoretical mechanical properties of MXenes, such as Young's modulus [107]. The exceptional mechanical performance makes MXene films well-suited as current collector as well as active material in energy storage devices [108]. Further, there has been a growing demand for small and portable "micro-electronic" system devices where MXenes have shown significant advancements [109, 110]. Less than 5% of the extensive research conducted on MXenes since their discovery has been devoted to investigating their mechanical and tribological properties [111]. The detailed mechanical proprieties and tribological properties have been summarized previously [111].

### Diversity

The MXene family offers a wide range of possibilities due to the combination of more than a dozen transition metal elements denoted as "M" and adjustable surface functional groups denoted as "T*x*." This diversity is further enriched by various processing steps that enable the construction of different macroscopic architectures of MXene. Examples of such architectures include powder, slurry, free-standing films, aerogels, hydrogels etc. [6, 57, 112]. Additionally, the morphology and structure of MXene can be modified and controlled, allowing further diversification. Moreover, based on the choice of the transition metal "M," MXene can be transformed into various derivatives through processes such as sulfurization, calcination, nitridation, selenization, sulfurization, and chlorination [113]. These transformations expand the variety of MXene-based materials even further, offering a broad range of possibilities for the development of novel MXene derivatives with unique properties for versatile applications.

### Surface Chemistry

Density functional theory (DFT) studies on MXenes have shown that MXene structures are fully terminated with functional groups. A greater negative energy value suggests a stronger bonding between the surface termination groups and the transition metals in MXenes [100]. The combinations of surface functional groups on MXene depend on the specific preparation methods and post-processing routes. Using HF or in situ HF-forming etching methods results in MXene surfaces possess the termination groups such as –O, –OH, and –F [6, 32]. Molten salt etching methods can introduce various functional groups like –Cl, –S, –Te, –Br, and –I [114]. Furthermore, post-thermal treatment can lead to the formation of –N groups [115]. These functional groups play a significant role in determining the physical and chemical performance of MXene. Further, the surface terminations of MXenes play a crucial role in determining their physical and chemical properties. The specific types and positions of surface terminations have a significant impact on various features of MXenes. Altering the surface chemistry of MXenes can have a profound effect on their electrochemical properties. Further, it is possible to obtain enhanced electrochemical performance by tailoring the surface terminations, in energy storage systems [100, 116, 117]. The presence of highly electronegative functional groups enables the formation of advanced MXene-based materials through electrostatic interactions with materials possessing a positively charged surface.

MXene also possesses other interesting properties, such as thermal, chemical, and optical [58, 118, 119]. All these advantages of MXenes such as a 2D structure, high electrical conductivity, mechanical strength, chemical stability, tunable surface chemistry offer a wide range of compositions, energy storage capabilities, and versatile applications [120–123]. These properties make MXenes highly promising materials for numerous technological applications and research endeavors.

## M_4_X_3_ MXenes for Energy Storage Applications

MXenes exhibit a distinctive combination of metallic conductivity and hydrophilicity, making them highly attractive as electrode materials for supercapacitors [124]. Their metallic conductivity enables efficient charge transport, while their hydrophilic nature facilitates electrolyte penetration and ion diffusion, leading to enhanced electrochemical performance. Herein, the recent advancements on Mono-M and DTM MXenes have been discussed for SCs.

### Ta- and Ta–Ti-based M_4_X_3_ MXenes

**Fig. 4:**
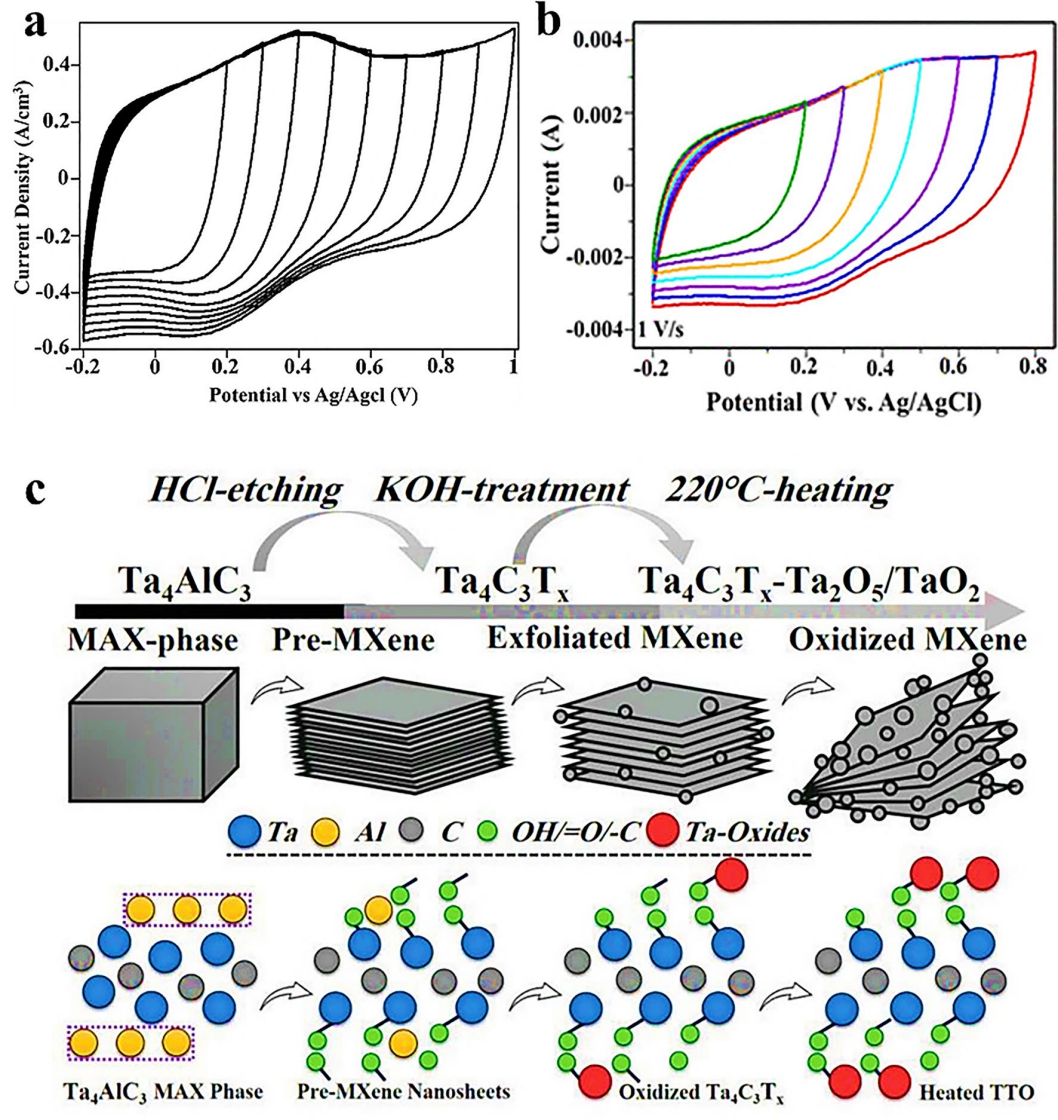
**a** CVs at different potential windows at a scan rate of 1 V s^−1^. Reproduced with permission from Ref. [72]. Copyright 2019, Elsevier; **b** CVs of MXene-Ti_*x*_Ta_4–*x*_C_3_ at different potential windows at a scan rate 1 V s^−1^. Reproduced with permission from Ref. [125]. Copyright 2023, ACS Publications; and **c** step-by-step synthesis and schematic model and stoichiometry of TTO hybrid structure for the fluorine-free conversion of the Ta_4_AlC_3_ MAX phase to surface-modified Ta_4_C_3_T_*x*_ MXene nanosheets decorated with tantalum oxide nanoparticles. Reproduced with permission from Ref. [127]. Copyright 2021, Wiley

MXenes have demonstrated excellent performance as electrode materials, offering their potential for improving the energy storage capabilities of aqueous supercapacitors [68]. Recently, Syamsai et al. [72] reported Ta_4_C_3_ MXene synthesized through HF etching route. The Ta_4_C_3_ MXene involves the removal of the aluminum (Al) layer from its MAX phase precursor. The electrochemical performance of Ta_4_C_3_ MXene was evaluated using cyclic voltammetry (CV) in a 0.1 M H_2_SO_4_ electrolyte under standard conditions. The CV measurements were conducted at various potential windows, ranging from − 0.2 to 1 V at a scan rate of 1 V s^−1^ in the three-electrode system. The CV curves demonstrated that the synthesized Ta_4_C_3_ MXene exhibited electrical double-layer capacitor (EDLC) behavior with quasi-pseudocapacitive characteristics, as shown in Fig. 4a. The same group reported double-ordered titanium tantalum carbide MXene nanosheets, based on Ti_x_Ta_4–x_C_3_ synthesized through same approach [125]. Comprehensive investigations involving phase, structural, and vibrational analyses were explored to confirmed the successful synthesis of MXene. The studies further revealed that the MXene layers exhibited a hexagonal crystal system. A specific capacitance of 200 F g^−1^ was achieved at a scan rate of 10 mV s^−1^ in two-electrode setup by using 1 M H_2_SO_4_. The CVs of the Ti_x_Ta_4–x_C_3_ were recorded at various potential windows, ranging from − 0.2 to + 0.8 V, using a scan rate of 1 V s^−1^ as shown in Fig. 4b. Such electrochemical measurement allowed the performance of the MXene material within the specified potential range. The CV curves provided insights into the redox processes and capacitance properties of Ti_x_Ta_4–x_C_3_, contributing to a better understanding of its electrochemical performance. Lopez et al. [126] combined both first-principles calculations and experimental measurements on the lithiation process in Ti_4_C_3_ and Ti_2_Ta_2_C_3_ MXene electrode materials. The findings indicate the successful synthesis of Ti_2_Ta_2_C_3_ MXene with an interlayer distance of 0.4 nm. The study demonstrated that the double-ordered Ti_2_Ta_2_C_3_ MXene has the ability to store four times the amount of lithium compared to the pristine Ti_4_C_3_ MXene. This enhancement in lithium storage capacity highlights the potential of Ti_2_Ta_2_C_3_ MXene for advanced energy storage applications.

In another study related to Ta metal, Rafieerad et al. [127] introduced a novel hybrid material composed of Ta_4_C_3_T_*x*_ MXene-tantalum oxide (TTO) as supercapacitor electrodes. The step-by-step schematic illustrations for the synthesis and functionalization of the mixed-dimensional TTO nanocomposite is shown in Fig. 4c. An innovative fluorine-free etching method was used to prepare a TTO hybrid nanostructure from MAX phase. Such approach not only provides a more environmentally friendly alternative but also enables the controlled synthesis and functionalization of the TTO hybrid nanostructure. This hybrid structure demonstrated a surface area that was 20% higher than oxidized Ta_4_C_3_T_*x*_. As a result, the hybrid material exhibited a volumetric capacitance of 447 F cm^−3^ at a scan rate of 1 mV s^−1^. This finding highlights the potential of the Ta_4_C_3_T_*x*_ MXene-TTO hybrid as a compatible and high-performance material for supercapacitor applications.

### Nb-based M_4_X_3_ MXenes

**Fig. 5:**
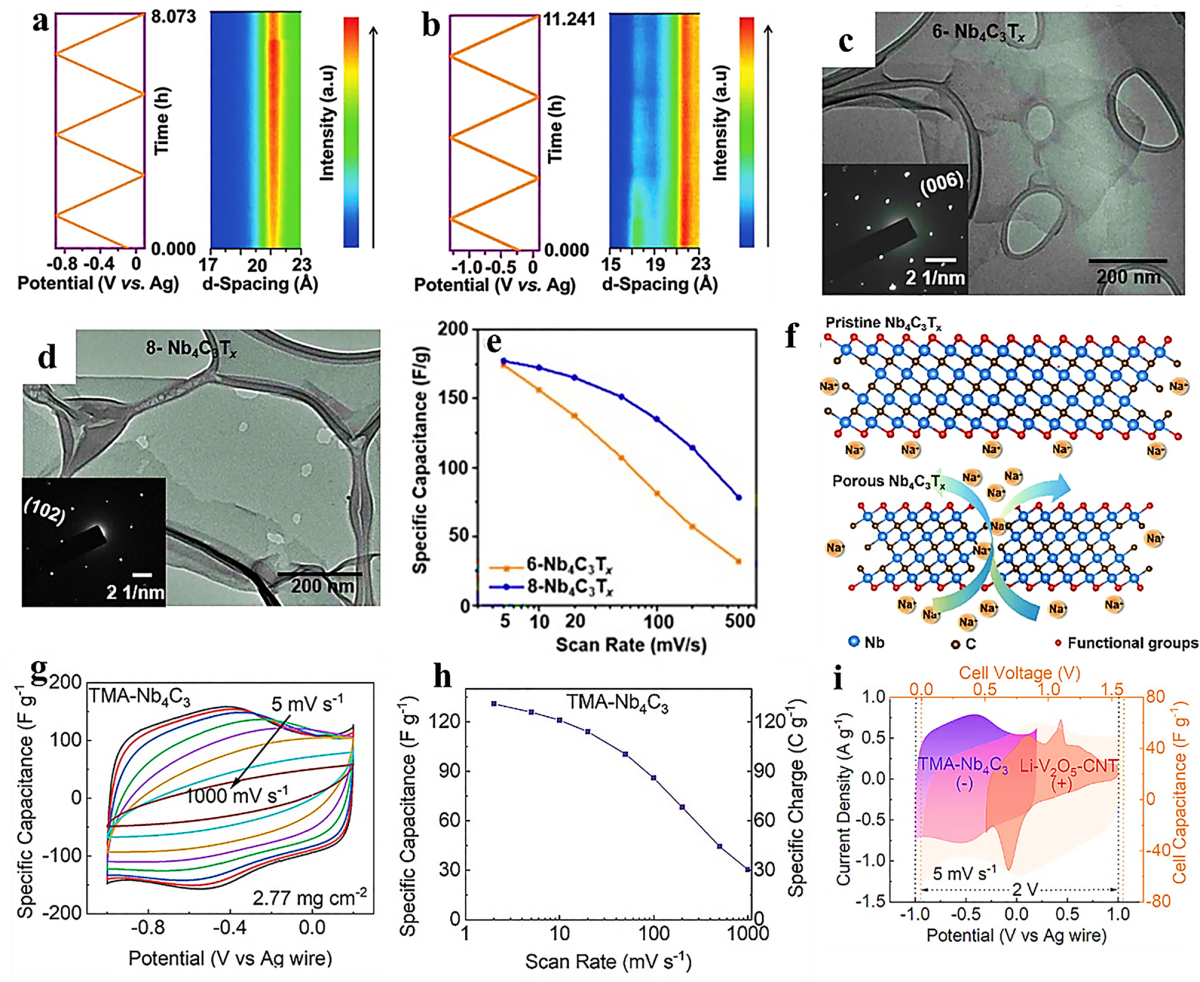
In-situ XRD patterns during electrochemical cycles in **a** 1 m H_2_SO_4_ and **b** 1 m MgSO_4_. Reproduced with permission from Ref. [128]. Copyright 2020 Wiley: TEM images of **c** 6-Nb_4_C_3_T_*x*_, **d** 8-Nb_4_C_3_T_*x*_ flakes. Inset shows SAED patterns, **e** variation of specific capacitance versus scan rate, and **f** schematic illustrating transport of electrolyte ions through Nb_4_C_3_T_*x*_ layers and ion diffusion pathways between MXene sheets and across a Nb_4_C_3_T_*x*_ flake with a pinhole. Reproduced with permission from Ref. [98]. Copyright 2022, Elsevier. **g** CVs of three-electrode cells containing a TMA-Nb_4_C_3,_ **h** calculated specific capacitances of the TMA-Nb_4_C_3_ electrode as a function of scan rate, and **i** CVs of three-electrode cells containing Li-V_2_C (− ve), Li-V_2_O_5_-CNT (+ ve) electrodes, and Li-V_2_C/Li-V_2_O_5_-CNT asymmetric cell at 5 mV s^−1^. Reproduced with permission from Ref. [67]. Copyright 2023, Wiley

Another representative M_4_X_3_ MXene, Nb_4_C_3_T_*x*_ has garnered attention across various fields due to its excellent conductivity and mechanical strength. Its unique combination of properties makes Nb_4_C_3_T_*x*_ a highly promising material for diverse applications, including energy storage, electronics, and structural engineering. The growing focus on Nb_4_C_3_T_*x*_ underscores its potential to drive significant advancements in various industries. Zhao et al. [128] successfully delaminated and transformed Nb_4_C_3_T_*x*_ MXene nanosheets into freestanding films, exhibiting an interlayer spacing of 1.77 nm. This interlayer spacing is larger compared to most previously reported MXenes. The freestanding Nb_4_C_3_T_*x*_ films were then tested as electrodes in supercapacitor, demonstrated high volumetric capacitance of 1075, 687, and 506 F cm^−3^ recorded in 1 M H_2_SO_4_, 1 M KOH, and 1 M MgSO_4_ electrolytes, respectively, at a scan rate of 5 mV s^−1^. To gain further insights into the structural changes occurring during the electrochemical charging process, an in-situ X-ray diffraction technique was employed as shown in Fig. 5a, b. The examination of the structural modifications of Nb_4_C_3_T_x_ in 1 M H_2_SO_4_ and 1 M MgSO_4_ electrolytes during the electrochemical charging process were carried out. During the electrochemical cycling process, it was observed that there was minimal change in the 21 Å interlayer spacing of the Nb_4_C_3_T_x_ MXene. In the three charge–discharge cycles, the (002) peak associated with the interlayer spacing which did not exhibit significant movement in 1 M H_2_SO_4_ (Fig. 5a). This observation suggests that the interlayer spacing of 21 Å in Nb_4_C_3_T_x_ MXene is sufficiently large to accommodate the intercalation of H^+^ ions without causing lattice expansion. A similar phenomenon is observed in Fig. 5b, where the interlayer spacing of Nb_4_C_3_T_x_ MXene in 1 M MgSO_4_ solution is as high as 21.5 Å (2θ = 3.8°–5.8°). This suggests that the space between the MXene layers is ample to accommodate the insertion/deinsertion of cations. The stable interlayer spacing indicates that the MXene structure effectively accommodated the necessary structural changes associated with the electrochemical reactions during cycling, ensuring the stability and durability of the material. This behavior further supported the suitability of Nb_4_C_3_T_x_ MXene for ion storage applications, as the interlayer spacing remains largely unchanged, allowing for efficient ion diffusion and reversible electrochemical processes. However, in the case of Mg^2+^ ions, their larger radius compared to H^+^ ions allow them to gradually remove the TMAOH intercalant after numerous cycles. This results in a decrease in capacitance retention over time. Overall, the stable interlayer spacing in Nb_4_C_3_T_*x*_ MXene enables efficient intercalation of H^+^ ions, while the larger size of Mg^2+^ ions can lead to the removal of intercalants, affecting the capacitance retention over extended cycling. The capacitive performance of Nb_4_C_3_T_*x*_ MXene in neutral aqueous electrolytes has been previously reported as moderate, with limited efforts made to enhance it.

Zhao et al. [98] reported a novel method to enhance the capacitive performance by introducing nanopores (pinholes) into Nb_4_C_3_T_*x*_ flakes through controlled etching time (6, 8, 10 days). The transmission electron microscopy (TEM) results (Fig. 5c, d) revealed that both samples exhibited transparent lamellar structures. The selective area electron diffraction (SAED) pattern displayed a distinct hexagonal structure, confirming the successful etching and delamination processes. In comparison to the smooth surface of the 6-Nb_4_C_3_T_*x*_ sample, the 8-Nb_4_C_3_T_*x*_ sample exhibited irregular pinholes on its surface. These pinholes were visually evident and indicated the introduction of nanopores in the material through the etching process. The presence of these pinholes suggested that the etching process was effective in generating the desired porous structure within the Nb_4_C_3_T_*x*_ MXene, potentially leading to improved ion diffusion pathways and enhanced electrochemical performance. The etching process conducted for a duration of 10 days resulted in excessive flake damage, rendering the material fragile. Figure 5e provides a summary of the gravimetric capacitance of both the 6-Nb_4_C_3_T_*x*_ and 8-Nb_4_C_3_T_*x*_ films at various scan rates which were calculated based on CV curves in 1 M Li_2_SO_4_ medium. Both the 6-Nb_4_C_3_T_*x*_ and 8-Nb_4_C_3_T_*x*_ films exhibited capacitance values of 174 F g^−1^ (490 F cm^−3^) and 177 F g^−1^ (equivalent to 485 F cm^−3^), respectively, at a scan rate of 5 mV s^−1^. Interestingly, when the scan rate is increased to 500 mV s^−1^, the capacitance of the 8-Nb_4_C_3_T_x_ film reaches 78 F g^−1^ (214 F cm^−3^). This value is nearly 2.4 times higher than that of the 6-Nb_4_C_3_T_*x*_ film, which exhibits a capacitance of around 32 F g^−1^ (90 F cm^−3^) under the same scan rates. The resulting holey Nb_4_C_3_T_x_ MXene exhibited a significant improvement in rate capability not only in 1 M Li_2_SO_4_ but also 1 M Na_2_SO_4_. This significant improvement in capacitance for the 8-Nb_4_C_3_T_*x*_ film suggested that the introduction of pinholes through the etching process enhances the rate capability of the material. A schematic representation (Fig. 5f) of the electrode–electrolyte interface incorporating the findings from the aforementioned analysis. This schematic illustrated the impact of pinholes on the ion transport mechanism in MXene films. For the Nb_4_C_3_T_*x*_ MXene without pinholes, ions in the electrolyte predominantly traverse through the gaps between the 2D layers. However, this transmission path is relatively long, potentially leading to slower ion diffusion and limiting the electrochemical performance. In contrast, the presence of pinholes in the Nb_4_C_3_T_*x*_ MXene allows ions to not only move through the gaps between the layers but also more rapidly access the interior of the material through the pores. This additional pathway provided by the pinholes effectively shortens the ion transport distance, facilitating faster ion diffusion and leading to improved electrochemical performance, particularly at higher scan rates. The introduction of pinholes in Nb_4_C_3_T_*x*_ MXene represents a promising approach to boost the capacitive performance of MXene materials in neutral aqueous electrolytes.

Recently, Mohit et al. [67] studied the electrochemical performance of TMA-Nb_4_C_3_ in 5 M LiCl. The CV results (Fig. 5g) within a potential window of − 1 to + 0.2 V of the TMA-Nb_4_C_3_ electrode were studied at various scan rates ranging from 5 to 1000 mV s^−1^. The CV curves indicate the pseudocapacitive-behavior of the electrode. At lower scan rates, the CV curves show the presence of a pair of broad peaks in both the anodic and cathodic scans. These peaks were indicative of the redox reactions associated with the pseudocapacitive-behavior of the electrode material. The specific capacitance of the TMA-Nb_4_C_3_ electrode reached a maximum value of 131 F g^−1^ at a scan rate of 2 mV s^−1^. The detailed specific capacitances at various scan rates have been illustrated in Fig. 5h. To expand the voltage window, an asymmetric cell using TMA-Nb_4_C_3_ (− 1.0 to 0.2 V) and Li-V_2_O_5_-CNT (− 0.3 to 1.0 V) electrodes were used to achieve favorable electrochemical performance for practical applications. A stable voltage window of 1.6 V was obtained which was lower than theoretical value. This discrepancy was due to the presence of overlapped junction regions in both the negative and positive electrodes (Li-V_2_O_5_-CNT and TMA-Nb_4_C_3_, respectively) within the TMA-Nb_4_C_3_/Li-V_2_O_5_-CNT asymmetric cell as shown in Fig. 5i. Despite this limitation, the asymmetric cell exhibited a capacitance of approximately 29 F g^−1^ at 1 A g^−1^, demonstrating its potential for energy storage applications. Additionally, the asymmetric cell demonstrated excellent cyclability, with a high retention of approximately 95% after 10,000 cycles, indicating its long-term stability and durability.

### V-based M_4_X_3_ MXenes

**Fig. 6:**
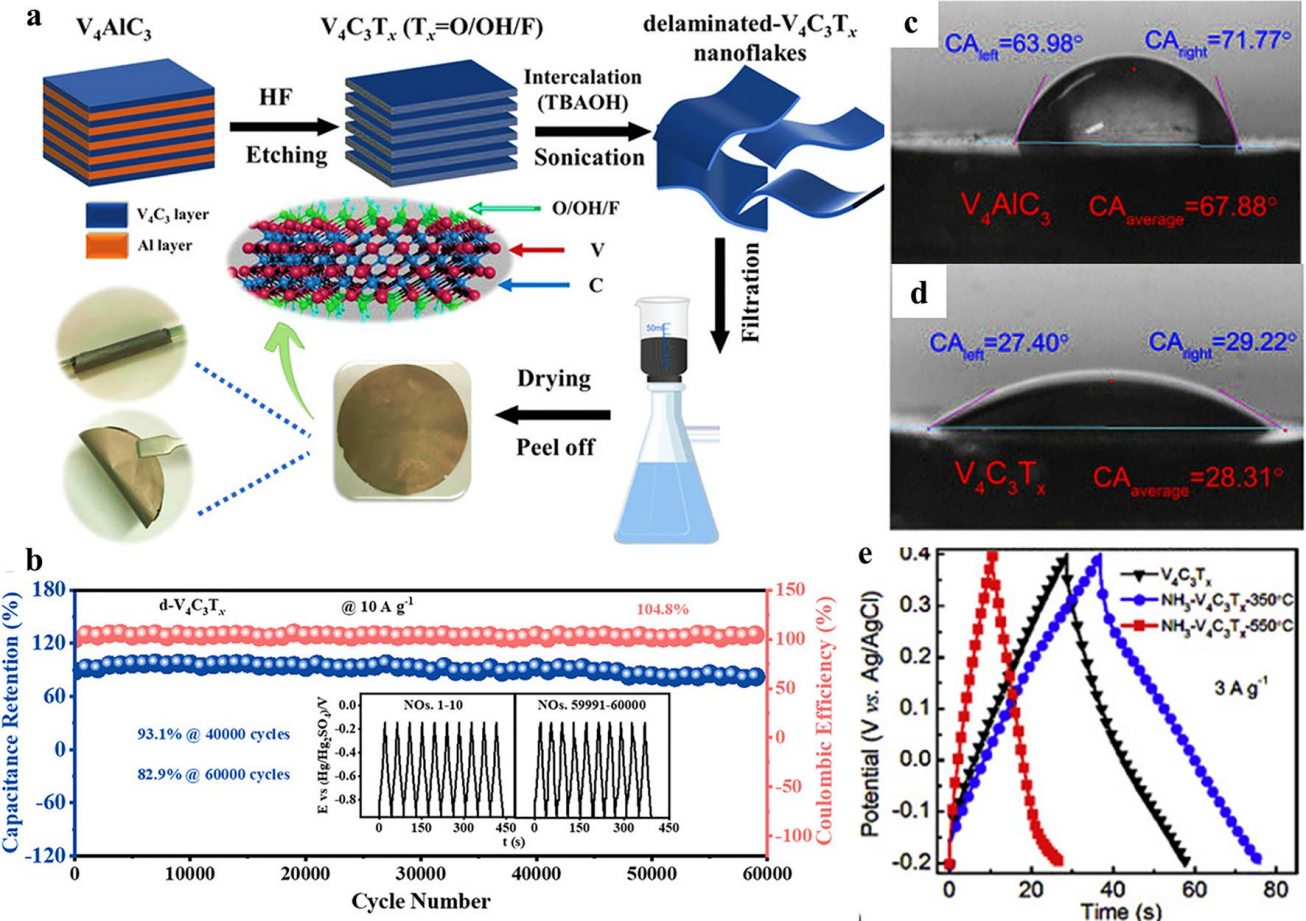
**a** Preparation schematic for the d-V_4_C_3_T_*x*_ film and **b** the d-V_4_C_3_T_x_ film for 60,000 cycles (the inset shows the charge–discharge profiles from the first to 10th cycles and the last ten cycles for the 60,000th cycles). Reproduced with permission from Ref. [96]. Copyright 2022, Wiley. Photographs of the water droplet shape with the contact angle (CA) on cold-pressed free-standing discs of the ball-milled **c** V_4_AlC_3_ and **d** V_4_C_3_T_*x*_. Reproduced with permission from Ref. [68]. Copyright 2019, Elsevier. **e** GCD curves of the V_4_C_3_T_*x*_, NH_3_-V_4_C_3_T_*x*_-350 °C and NH_3_-V_4_C_3_T_*x*_-550 °C at 3 A g^−1^. Reproduced with permission from Ref. [69]. Copyright 2019, Elsevier

The V_4_C_3_ MXene possesses expanded interlayer spacing and abundant redox active sites, indicating its potential as an electrode material. The enlarged interlayer spacing V_4_C_3_T_*x*_ MXene allows easier ion intercalation and promotes efficient charge transport within the material. Additionally, the presence of numerous redox active sites provides additional pathways for charge transfer, further enhancing its electrical conductivity. Bin et al. [96] reported V_4_C_3_T_*x*_ MXene to tackle with the stability and poor performance of compactly stacked layers-based electrode materials. The MAX phased of V-based MXene was transformed into multi-layered V_4_C_3_T_*x*_ MXene, and then further delaminated to single layer nanoflakes (d-V_4_C_3_T_*x*_) using tetra-n-butylammonium hydroxide (TBAOH). These delaminated nanoflakes had the capability to self-assemble into a flexible film without the need for a binder material. Figure 6a illustrated the schematic representation of the preparation process for the flexible and free-standing d-V_4_C_3_T_*x*_ film. The resulting d-V_4_C_3_T_x_ film exhibits a significant interlayer spacing of 2.1 nm. This enlarged spacing plays a crucial role in facilitating the diffusion of ions, allowing for reversible intercalation and deintercalation processes without causing damage to the layered structure. As a result, the d- d-V_4_C_3_T_*x*_ film demonstrated excellent electrochemical performance, presenting its superior capabilities in energy storage and other electrochemical applications. The excellent cycle performance of the d-V_4_C_3_T_*x*_ film electrode was studied through a repeated GCD cycles. The film electrode was subjected to 40,000 cycles at a high current density of 10 A g^–1^ and a wide potential window ranging from − 0.95 to − 0.15 V, retaining 93.1% of the capacitance (Fig. 6b). Even after an extended duration of 60,000 cycles, the capacitance retention remained 82.9%. Furthermore, the GCD cycles of the first and last ten cycles (60,000th cycle) are shown in the inset of Fig. 6b. It was evident that the GCD curves before and after the cycles were nearly identical, indicating no significant deformation or deterioration in the electrode's performance. This observation further confirms the outstanding cycle reversibility demonstrated by the flexible d-V_4_C_3_T_*x*_ film electrode.

Teng et al. [38] studied V_4_C_3_T_*x*_ MXenes which exhibited a notable disparity in their performance between acidic and basic electrolytes. In acidic electrolytes, V_4_C_3_T_*x*_ demonstrated a superior capacitance of 284 F g^–1^, indicating its excellent energy storage capabilities. However, in basic electrolytes, its performance was comparatively moderate. V_4_C_3_T_*x*_ MXenes showed significant electrochemical stability even after 60,000 cycles, highlighting maintained structural integrity these MXenes. In addition to large interlayer spacing, high specific surface areas and pore volumes, good conductivity etc. the hydrophilicity was also crucial factor to be measured when using MXenes as electrode materials for SCs. Thus, in the context of SC electrodes operating with aqueous electrolytes, the contact angle (CA) with water is a critical parameter to be considered. Wang et al. [68] measured the CA of the V_4_C_3_T_x_ MXenes and its MAX phase surfaces (Fig. 6c, d). In the case of V_4_C_3_T_*x*_ MXenes, the average contact angle was determined to be 28.31° which was much lower than the MAX phase V_4_AlC_3_ (67.88°), suggesting a greatly enhanced hydrophilic behavior. In other words, the as-synthesized V_4_C_3_T_*x*_ MXene exhibits a stronger attraction to water, indicating a higher affinity for aqueous electrolytes, which was desirable for efficient supercapacitor performance. When employed as a supercapacitor electrode, the V_4_C_3_T_*x*_ MXene material exhibited higher capacitance of approximately 209 F g^−1^ at a scan rate of 2 mV s^−1^. The MXene electrode demonstrated a long-term cyclic stability of 97.23% after 10,000 cycles at a current density of 10 A g^−1^. This signified the ability of MXenes and made it a reliable and durable option for SC applications in 1 M H_2_SO_4_ electrolyte. Li et al. [69] improved the V_4_C_3_T_*x*_ MXenes performance through nitrogen doping at different annealing temperatures. The concentration of nitrogen in V_4_C_3_T_*x*_ MXene was controlled and optimized by annealing temperatures. The MXene treated at 350 °C (NH_3_-V_4_C_3_T_*x*_-350 °C) exhibited a higher specific capacitance of 210 F g^−1^ compared to pristine and other doped V_4_C_3_T_*x*_ MXene at a scan rate of 10 mV s^−1^ in 1 M H_2_SO_4_ electrolyte (Fig. 6e). Such study indicated that the nitrogen-doped MXene electrode has an improved ability to store electrical charge, leading to enhanced energy storage performance. Additionally, NH_3_-V_4_C_3_T_*x*_-350 °C also exhibited excellent cycling stability, indicating that its electrochemical performance remains consistent over multiple charge–discharge cycles.

### Mo-Ti-based M_4_X_3_ MXenes

**Fig. 7:**
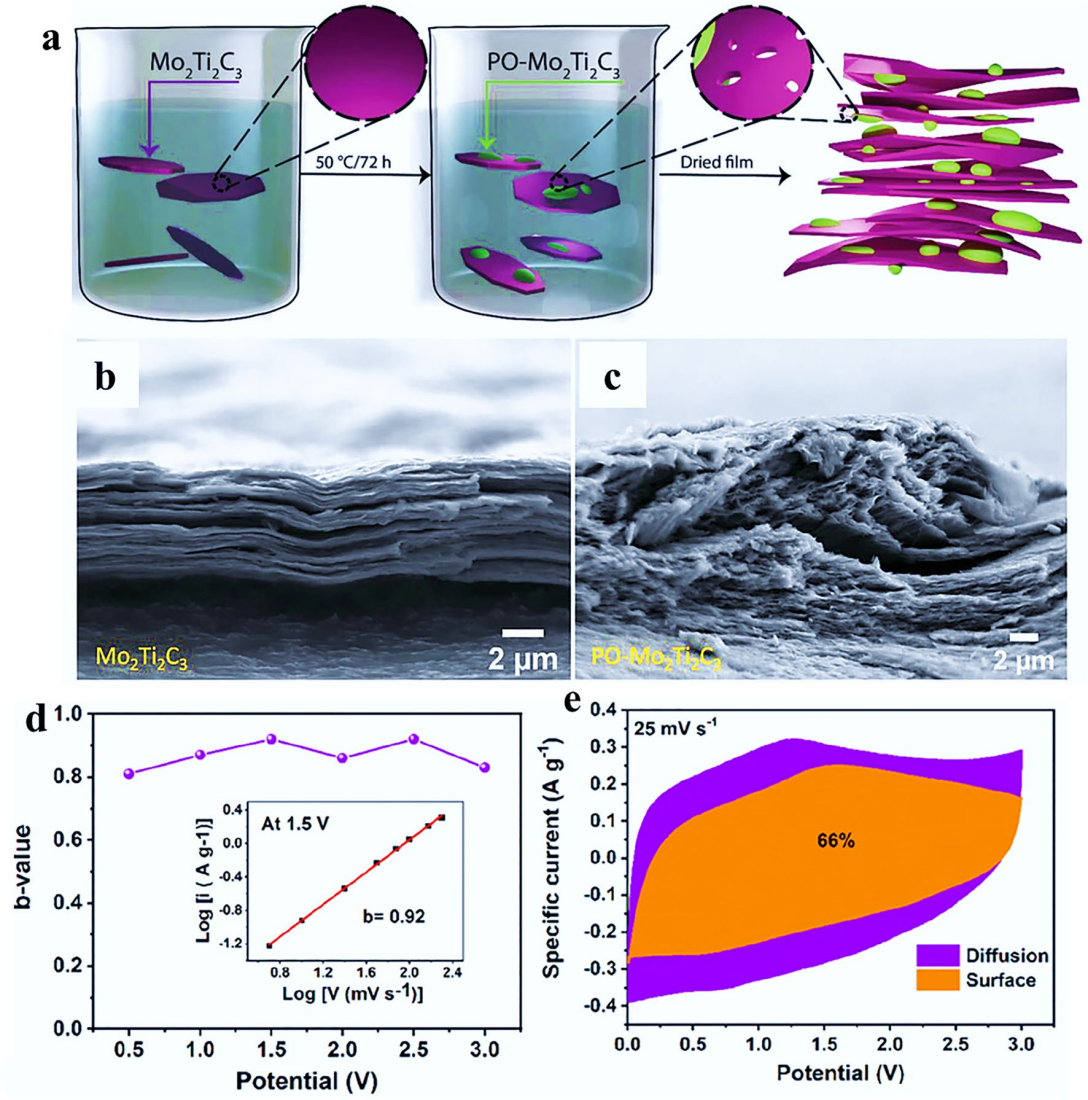
**a** Schematic illustration of the synthesis of partially oxidized Mo_2_Ti_2_C_3_ (PO-Mo_2_Ti_2_C_3_), cross-sectional SEM images of **b** Mo_2_Ti_2_C_3_ and **c** PO-Mo_2_Ti_2_C_3_ free-standing MXene films. Reproduced with permission from Ref. [129]. Copyright 2023, Wiley. **d** Variation of *b*-values as a function of potential for the anodic scan. The inset shows the power–law dependence of the peak current at scan rates from 5 to 200 mV s^–1^. **e** Percentage of the surface-controlled and diffusion-controlled area in the CV curve at a scan rate of 25 mV s^–1^ for f-Mo_2_Ti_2_C_3_. Reproduced with permission from Ref. [130]. Copyright 2022, ACS Publications

The greater the range of chemical compositions and structural complexities found in double transition metal MXenes. Thus, the exploration of mixed metallic MXenes, specifically out-of-plane ordered Mo_2_Ti_2_C_3_, in energy storage applications has been a good choice which is also less explored due to the lack of redox activity in many electrolytes. To address these limitations and enhance the electrochemical properties, a promising strategy involves simultaneous structural modifications and the induction of intercalation pseudocapacitance in neutral electrolytes. By implementing such approach, it is possible to enhance the electrochemical performance of mixed metallic MXenes and unlock their potential for energy storage applications. Mohit et al. [129] presented a simple method for synthesizing partially oxidized Mo_2_Ti_2_C_3_ MXene, noted as PO-Mo_2_Ti_2_C_3_, which exhibits enhanced charge storage capability. A straightforward and efficient method was used for producing partially oxidized PO-Mo_2_Ti_2_C_3_ through thermal annealing of pristine Mo_2_Ti_2_C_3_ under mild conditions, as depicted in Fig. 7a. The structural analysis of PO-Mo_2_Ti_2_C_3_ reveals the presence of pinholes, high roughness, and oxide nanostructures on the surface of the MXene. The free-standing films of PO-Mo_2_Ti_2_C_3_ exhibit a rougher surface compared to smooth films of Mo_2_Ti_2_C_3_ which display a more ordered structure. The comparative SEM images of pristine Mo_2_Ti_2_C_3_ and PO-Mo_2_Ti_2_C_3_ are depicted in Fig. 7b, c. The roughness observed in the PO-Mo_2_Ti_2_C_3_ films can be attributed to the formation of the oxide layer upon heating. This structural difference between the two types of films highlights the impact of thermal oxidation on the surface characteristics and ordering of the MXene material.

These oxide nanostructures act as spacers, preventing the restacking of MXene sheets and facilitating improved intercalation of Li + ions between the layers of PO-Mo_2_Ti_2_C_3_ in a 5 m LiCl electrolyte. As a result of the enhanced intercalation was observed and several improvements compared to pristine Mo_2_Ti_2_C_3_. The charge storage capacity, cycle life, and Coulombic efficiency were all enhanced in PO-Mo_2_Ti_2_C_3_. The formation of oxide nanostructures when Mo_2_Ti_2_C_3_ was observed when subjected to thermal oxidation by using various optical, structural, and spectroscopic analyses. This indicates the potential of our approach to enhance the electrochemical performance of Mo_2_Ti_2_C_3_-based materials for energy storage applications.

The energy density of supercapacitors is often limited by the narrow potential window in aqueous electrolytes. Ionic liquid electrolytes offer a higher potential window and superior specific energy but can be challenging due to slow ion transport and difficult intercalation caused by their larger ion size. Therefore, it is desirable to investigate MXenes with larger interlayer-spaced (*d*-spaced) structures that can facilitate the intercalation and deintercalation of larger ions. Gandla et al. [130] presented the Mo_2_Ti_2_C_3_ MXene free-standing film electrode in a supercapacitor. 1M 1-ethyl-3-methylimidazolium bis(trifluoromethylsulfonyl)imide (EMIMTFSI) in acetonitrile electrolyte was utilized, which provides a wider potential window compared to aqueous electrolytes. The Mo_2_Ti_2_C_3_ MXene electrode demonstrates excellent performance in this system, benefiting from the larger interlayer spacing that facilitates the intercalation and deintercalation process of the larger ions. The b-values ranged from 0.82 to 0.93 were obtained at various potentialsas depicted in Fig. 7d. These *b* values indicate the dominance of the surface-controlled capacitive process over intercalation pseudocapacitive. The surface-controlled capacitive current was approximately 66% of the total current at a scan rate of 25 mV s^−1^ for Mo_2_Ti_2_C_3_ MXene (Fig. 7e). A significant increase was observed in the capacitive contributions at the higher scan rates, suggesting rapid charge transfer on the surface area. Further, Pinto et al. [131] have successfully synthesized and characterized four different compositions of Mo_x_V_4−x_C_3_ with x values of 1, 1.5, 2, and 2.7. This study demonstrated that by adjusting the Mo: V ratio, surface terminations (O: F ratio) can be modified which tune the electrical and electrochemical properties of the resulting MXenes. Notably, the Mo_2.7_V_1.3_C_3_ composition exhibited a volumetric capacitance up to 860 F cm^−3^, and displayed high electrical conductivity of 830 S cm^−1^ at room temperature. Furthermore, these solid solution MXenes exhibited a wider range of positive potentials compared to other MXenes. Authors used Mo_2.7_V_1.3_C_3_ as a positive electrode and combined it with a well-studied Ti_3_C_2_ MXene negative electrode to produce an all-MXene supercapacitor, demonstrating the potential of utilizing these tailored MXene compositions for high-performance energy storage applications. Further, Wang et al. [132] used a wet chemical etching method with hydrofluoric acid (HF) and successfully produced a solid-solution V_4−*y*_Cr_*y*_C_3_T_*x*_ (*y* = 0, 1, and 2) MXene derived from the solid-solution MAX phase. The addition of the Cr to second M-site metal brought a significant enhancement in the physiochemical properties of the V_4−*y*_Cr_*y*_C_3_T_*x*_ MXene, resulting improved electrochemical results. The electrochemical performance of such MXenes have been tabulated in Table 1.

**Table 1:** M_4_X_3_ MXene electrode and device electrochemical performance for supercapacitors

M_4_X_3_ MXene	Electrolyte	Electrode/device performance		References
Composition	Aqueous/organic	Specific capacitance	Cycling stability/Retention%	
Mo_2_Ti_2_C_3_	5 M LiCl	102 F g^−1^ at 5 mV s^−1^	10,000/85	[129]
PO-Mo_2_Ti_2_C_3_	5 M LiCl	132 F g^−1^ at 5 mV s^−1^	10,000/100	[129]
f-Mo_2_Ti_2_C_3_//f-Mo_2_Ti_2_C_3_	1 M EMIMTFSI	152 F g^−1^	5,000/86	[130]
Mo_2.7_V_1.3_C_3_	1 M H_2_SO_4_	300 F g^−1^	12,000/90	[131]
Zn//V_3_CrC_3_T_x_	3 M ZnSO_4_	397.5 F g^−1^	50,000/70.2	[132]
Ti_2.9_Nb_0.1_C_2_T_x_	1 M H_2_SO_4_	104 F cm^−3^ at 2 mV s^−1^	84,000/67.9	[133]
Ti_x_Ta_4–x_C_3_//Ti_x_Ta_4–x_C_3_	1 M H_2_SO_4_/PVA	200 F g^−1^	30 days/80	[125]
V_4_C_3_T_*x*_	1 M H_2_SO_4_	210 F g^−1^	10,000/96.3	[69]
V_4_C_3_T_*x*_	1 M H_2_SO_4_	330 F g^−1^	3,000/90	[134]
V_4_C_3_	1 M H_2_SO_4_	209 F g^−1^	10,000/97.23	[68]

Moreover, Huang et al. [71] reported the optimized synthesis strategy to obtain high-quality few-layer M_4_X_3_ MXenes (where M = V, Nb, Ta) that involves several key steps. These steps include precursor calcination, HF etching, intercalation, and exfoliation processes. By combining theoretical and computational analyses with experimental investigations, researchers can gain a comprehensive understanding of not only M_4_X_3_ MXenes but also other MXenes as well as their potential applications.

## Conclusion and Future Aspects

**Fig. 8:**
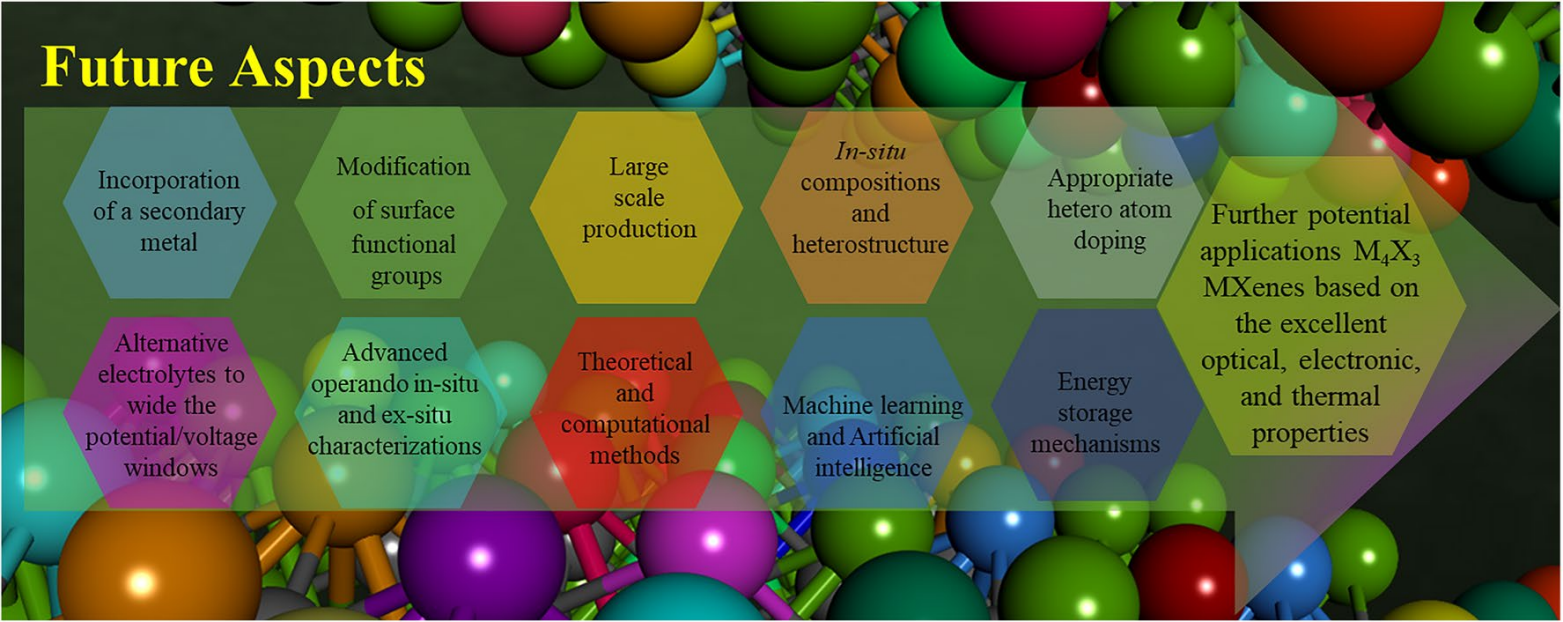
Future directions of M_4_X_3_ MXenes

In summary, this review highlights the recent advancements in M_4_X_3_ MXenes for supercapacitor electrode materials. The unique properties of 2D MXenes have also been discussed which offer several advantages over traditional materials as well as other MXenes. M_4_X_3_ have demonstrated excellent physicochemical properties, suggesting their potential for future advanced energy storage devices. However, there are still challenges to overcome in the research of M_4_X_3_-based electrode materials. Following futures aspects have been suggested:

- Incorporating a secondary metal along the early transitional metal group could further expand its properties to targeted applications. Introducing a secondary metal in MXene in the preparation stage of its initial MAX precursor can be beneficial. Apart from mono metal M_4_X_3_ MXene, it is important to explore the application of different compositions of double metal MXenes. Further, in the synthesis process, various surface functional groups can be introduced using different strategies for desired applications. For the discovery of new double transitional metal MXenes a new MAX phases are suggested to be studied.
- Effort should be directed towards the large scale the production of MXenes. However, it is essential to overcome challenges related to temperature regulation, mitigation of toxicity, and prevention of equipment corrosion.
- Indeed, exploring composite electrodes composed of MXenes holds great promise for energy storage applications. M_4_X_3_ MXenes can be further transformed to MXene in-situ composites and heterostructure without using other transition metal precursors [113]. Such heterostructured electrodes have the potential to harness the synergistic effects of the unique features offered by both 2D MXene and its composite materials. Further, the integration of hybrid structures could effectively enhance both electric double-layer capacitance (EDLC) and pseudocapacitance.
- Further potential applications M_4_X_3_ MXenes are suggested to be explored due to the excellent optical, electronic, and thermal properties.
- Surface engineering techniques, such as appropriate hetero atom doping and the synthesis of MXenes without particular terminations can be investigated to increase the interlayer spacing of MXene electrodes. This enhancement would enable improved electrochemical performance. A systematic exploration of functional groups and their impact on capacitance has the potential to open up new avenues for enhancing the performance of energy storage systems. Such investigation will help to find the materials with enhanced electrochemical behavior in energy storage applications.
- Alternative electrolytes to aqueous electrolytes with unique properties and wider electrochemical potential/voltage windows can be investigated to enable faster ion transport and create a safe and environmentally friendly environment for energy storage applications. Thus, exploring new electrolytes can potentially unlock new opportunities to enhance the overall performance of MXene materials in energy storage applications.
- It is evident that the complete covering or wrapping MXenes (especially M_4_X_3_ MXenes) with metal/metal oxide/metal sulfides are hindered due to the lack of flexibility in its structures. Such structural limitations effect the stability of the MXene-based electrode. To enhance the cycling stability, the construction of pillared structure with robust mechanical strength is highly recommended.
- Proper balance between the mechanical characteristics and the electrochemical properties is highly essential for any active materials in energy storage devices. In this aspect, defect-free MXenes (with less prone to oxidation) should be synthesized by following sophisticated synthetic routes.
- The current commercial current collectors still face several challenges. To overcome these limitations and achieve high-energy density in other energy storage devices such as batteries, exploring and designing innovative MXene based current could be a promising strategy. This approach might open up opportunities for advancing battery technology and improving overall battery performance.
- Advanced operando in-situ and ex-situ characterization techniques are indeed crucial for gaining a comprehensive understanding of the multi-functionalities of MXenes. This enables researchers to gain a thorough understanding of real-time observations and analyses of ion diffusion, charge transfer kinetics, and structural changes in MXene.
- Theoretical and computational methods are suggested to be employed. These methods play a significant role in understanding the properties and energy storage mechanisms of novel MXene and its hybrid materials.
- In MXenes with M_4_X_3_ composition and thicker layers, the M atoms in the inner layers are generally considered to be electrochemically inactive, in contrast to the M atoms in the outer layers. This should be further investigated to enhance the overall performance.
- The combination of advanced characterization techniques, theoretical/computational methods, and also machine learning and artificial intelligence offer a powerful approach for advancing the understanding and development of MXenes, ultimately leading to the design of improved energy storage devices.
- Green and cost-effective synthetic approaches for MXenes are highly essential in the light of current global situation and future electronics world. The detailed future suggestions are summarized in Fig. 8.

In summary, M_4_X_3_ MXene-based supercapacitors offer an exciting and promising properties to be explored. The purpose of this review is to give researchers a common platform to obtain information about the properties of M_4_X_3_ and its applications in supercapacitors. Additionally, this review offers suggestions to be considered in the future applications.
